# A new antioxidant made from a pterostilbene functionalized graphene nanocomposite as an efficient treatment for dry eye disease

**DOI:** 10.3389/fchem.2022.942578

**Published:** 2022-08-24

**Authors:** Mimi Lin, Xueqin Sun, Sihao Ye, Youyi Chen, Jing Gao, Feng Yuan, Na Lin, Tom Lawson, Yong Liu, Ruzhi Deng

**Affiliations:** ^1^ Laboratory of Nanoscale Biosensing and Bioimaging (NBAB), School of Ophthalmology and Optometry, School of Biomedical Engineering, Wenzhou Medical University, Wenzhou, Zhejiang, China; ^2^ State Key Laboratory of Ophthalmology, Optometry, and Vision Science, Eye Hospital, Wenzhou Medical University, Wenzhou, Zhejiang, China; ^3^ School of Mathematical and Physical Sciences, ARC Centre of Excellence for Nanoscale Biophotonics (CNBP), Macquarie University, Sydney, NSW, Australia

**Keywords:** dry eye, pterostilbene, functional graphene nanocarriers, human corneal epithelial cells, reactive oxygen species

## Abstract

Dry eye disease is a common condition that affects the eyes. It is caused by problems with the tear film and the tear dynamics. Dry eye can be caused by an increase in the amount of reactive oxygen species (ROS) in the corneal epithelium. The treatment for dry eye typically focuses on relieving the uncomfortable symptoms by using eye drops such as artificial tears, antibiotics, and by using anti-inflammatory/immunosuppressive agents such as cyclosporine, and lifitegrast. However, the recovery of patients with dry eye can take several years particularly if the symptoms are severe. This is because the present treatment approaches for dry eye are not based on its cause, e.g., the oxidative stress arising from the rapid increase in ROS. This work describes a new type of antioxidant made from pterostilbene (PS) and carboxyl-chitosan modified graphene (CG). The use of a hydrophilic two-dimensional CG nanosheet to improve the properties of PS is reported. Superior enhanced properties including better cellular permeability, long sustained release period (over 30 h), and antioxidant properties, were realized by using PS-CG. A hyperosmotic (HS) damaged human corneal epithelial cell (HCEC) model was used for antioxidant tests. This model has an intracellular ROS level 4 times more than that of a control group. The ROS content was declined efficiently to the same amount as normal cells in the PS-CG treated HS group. There was a significant decline in the content of lactate dehydrogenase (LDH) and the apoptosis rate of HCEC in the PS-CG treated HS group when compared to that seen in the HS model. Real-time polymerase chain reaction (PCR) and western blots (WB) were used to understand the antioxidant mechanism of PS-CG. The results showed that the antioxidant was working by activating the Keap1-Nrf2-ARE signalling pathway. *In vivo* testing testing using a dry eye mouse model suggested that the PS-CG acted as an efficient antioxidant. More tear production and healthier corneal and conjunctival epithelial cells were achieved when PC-CG was applied to this model. The use of PS-CG could be a new strategy for treating dry eye and other ocular diseases caused by ROS.

## Introduction

Dry eye is a disease that makes your eyes feel dry and irritated. It’s a common problem, and about 11%–28% of people have it. Dry eye disorder is associated with the way tears are distributed on the surface of the eye. Dry eyes can cause symptoms like eye strain, a gritty feeling in the eye, and even visual impairment. Usually, dry eye is accompanied by increased osmotic pressure in the tear film and inflammation on the surface of the eye (Su and Chang, 2021; Kim et al., 2022; Talens-Estarelles et al., 2021). Severe dry eye always causes significant vision loss. Traditional clinical treatment for dry eye focuses on relieving its uncomfortable symptoms by the use of eye drops such as artificial tears, antibiotics, and anti-inflammatory/immunosuppressive agents such as cyclosporine, and lifitegrast (Wilson and Perry, 2007; Bron et al., 2017). It takes a long time for people with dry eye DE to recover, especially if they are severely ill. The problem is that there are too many reactive oxygen species (ROS) in the cells of the human corneal epithelial cells (HCEC), and this causes the dry eye (Lee et al., 2017). Some recent studies have shown that oxidative stress is a major cause of dry eye inflammation (Kojima et al., 2020; Wang et al., 2021). Markers of oxidative stress damage were found in the tears of people with dry eye (Cejková et al., 2007; Cejková et al., 2008; Choi et al., 2016; Navel et al., 2021). There is more oxidative stress (Jiao et al., 2022), or damage from things like pollution and sunlight, in their tears. Recently, it was suggested that the increased ROS activated the inflammasome NLRP3, which caused this immune-inflammatory response (Yuan et al., 2022; Wan et al., 2022).

This work shows that controlling the oxidative stress of HCEC cells could be a potential treatment for DE (dry eye). A new antioxidant nanocomposite has been created from pterostilbene and carboxyl-chitosan modified graphene. Pterostilbene (PS) is found in blueberries and is known for its antioxidant ability. This new nanocomposite has the potential to fight oxidation. PS is thought to be beneficial to many diseases, such as cardiovascular diseases, cancers, diabetes, neurological diseases, and fundus diseases (Li et al., 2016; Seen and Tong, 2018; Levenson, 2022). PS is a material that has potential for some medical applications, but it has some poor properties that limit how it can be used. This includes PS’s hydrophobicity, poor cellular permeability and retention capability on the ocular surface. In this work, carboxyl-chitosan functionalized graphene (CG) was used to address those properties. A previous study by the authors showed that CG is good for hydrophilicity and ocular biocompatibility, which means it is good for ophthalmic applications (Zou et al., 2020). Particularly, the long-range π-electron system structure of graphene-based materials (including CG) has shown a strong affinity to the electrons and free radicals such as ROS (Wang et al., 2020). The nitrogen-doping (which comes from the chitosan molecules) into the π-π conjugated carbon rings of CG in CG offers additional advantages for the catalytic reduction of oxygen and removal of ROS (Zhang et al., 2016; Deng et al., 2019).

This work reports on the creation of a new type of antioxidant that can be used to treat dry eye caused by oxidative stress. The as-prepared PS-CG had a good antioxidant effect because of the synergistic effects of PS and CG. The mechanism of the antioxidant effect is associated with the activation of the Keap1-Nrf2-ARE signalling pathway, which enhances the expression of various antioxidative enzymes and detoxification enzymes. This work suggests that certain proteins can help to prevent oxidative stress, which can lead to a dry eye disease. This could be a new way to treat ocular diseases in the future that are related to oxidative stress.

## Experimental procedure

### Materials and experimental equipment

The graphite powder flake was provided by Qinghai Haida Graphite Co., Ltd., Carboxylated chitosan was purchased from Shanghai Aladdin. PS and dimethyl sulfoxide (DMSO) were bought from Sigma-Aldrich. Human corneal epithelial cells (HCEC) were purchased from ATCC. Fetal bovine serum and Dulbecco’s Phosphate Buffered Saline (DPBS) were purchased from GIBCO. CCK-8 kits and Calcein-AM/PI kits were obtained from Dojindo. FITC Annexin V apoptosis kit was from Becton, Dickinson and Company (BD). BALB/C mice were obtained from Zhejiang Weitong Lihua Experimental Animal Technology Co., Ltd.

Experimental instruments used in this work include a QM-2SP12 planetary ball milling machine (Nanjing NanDa Instrument Plant, China), a Cary100 UV-visible Spectrophotometer (Agilent, Australia), a Multimode 8 Atomic Force Microscope (Bruker, German), a Nicolet 6,700 Infrared Spectrometer (Thermal Scientific, United States), an Invia Raman spectroscope (Renishaw, United Kingdom), a FL1000 Universal Gel Imager (Thermo Scientific, United States), an IX81 Fluorescence Inverted Microscope (Olympus, Japan), a Spectramax M5 (Molecular Devices, United States), a Cytoflex Flow Cytometry (Beckman Coulter, United States), an X50 Reverse Transcription PCR Instrument (Eppendorf, Germany), and an ABI QuantStudio 3 Real-time Quantitative PCR (ABI, United States).

### Synthesis of pterostilbene functionalized graphene nanocomposite

CG was prepared *via* an edge functionalized ball milling method (Zou et al., 2020). Typically, graphite powder and carboxyl chitosan were mixed at a mass ratio of 1:20 in a ball milling machine. The mixture was milled at 500 r/min for 4–8 h. DI water was used to transfer the mixture into a tube. The impurities that didn’t react were removed by a centrifugation machine that spun at 8,000–10,000 rpm, followed by a dialysis process using DI water. CG precipitate was obtained after centrifugation. This precipitate was dispersed in a DMSO solution. The CG/DMSO dispersion was mixed with a PS dissolved DMSO solution using a rotary shaker over 24 h. PS was covalently linked to CG *via* a π-π stacking interaction. The mixture was then centrifuged, and the precipitate was collected. This was dissolved in DI water to prepare a PS-CG dispersion.

### The pterostilbene release from the pterostilbene functionalized graphene nanocomposite

0.1% Tween 20 was added to 200 ml DI water at a pH of 7.2 in a beaker. The beaker was placed in a thermostatic shaker. 2 ml of 1.0 mg/mL PS-CG in a dialysis bag (molecular weight of 8,000 Da) was placed in the beaker. This process was completely carried out in the dark. 2 ml samples were taken from the beaker at regular intervals, and then 2 ml DI water (pH = 7.2) was added to the beaker to keep the volume the same. Samples were collected for detection using UV-vis spectrometry. The absorption peak of PS is at 325 nm. The released concentration of PS can be calculated from a standard UV absorption intensity at 325 nm vs. the concentration curve.

### *In vitro* experiments

Human corneal epithelial cells (HCEC) were used for *in vitro* experiments. A DMEM/F12 cell culture medium (containing 10% fetal bovine serum and 1% penicillin-streptomycin double resistance) was used for cell culture. Cells were incubated at 37°C, and with 5% CO_2_. A Cell Counting Kit-8 (CCK-8) test was used to see how many cells survived after being co-cultured with nanomaterials. The CCK-8 cell viability assay contained water-soluble WST-8. WST-8 can be reduced to a yellow formazan by dehydrogenase in the mitochondria of cells. The amount of the yellow formazan formed is proportional to the number of living cells present. The light absorption value measured at 450 nm counted the number of living cells indirectly by their reflection. Cells were seeded in a 96-well plate at a density of 5,000 per well. They were pre-cultured for 24 h. 10 μl CCK-8 solution and 100 μL cell culture solution were added to each well.

The treated cells were placed in a cell incubator for 2 h. The absorbance value at 450 nm was recorded by a microplate reader. The number of cells present is proportional to the absorbance value. The more cells there are, the higher the value becomes. The intracellular reactive oxygen species (ROS) content was measured by a 2,7-Dichlorodihydrofluorescein diacetate (DCFH-DA) probe. The DCFH-DA probe is not fluorescent.

The molecule can enter and penetrate the cell membrane. Once inside, it is hydrolyzed to DCFH. DCFH cannot penetrate the membrane, so it is located inside the cell. DCFH can be oxidized to fluorescent DCF by ROS. The intensity of green fluorescence from the DCF is proportional to the expression level of ROS. The excitation wavelength was 488 nm, while the emission wavelength was 525 nm.

An Annexin V-PI apoptosis detection kit was used to test for cell apoptosis or cell death. HCEC cells were seeded in a 6-well plate. After 24 h, Annexin V-FITC or PI, or a mixture of the two was added to the cell culture medium. In the experiment, the cells were placed in an incubator in the dark. The incubator was set to 37°C with 5% CO_2_. The cells were left in the incubator for 15 min. The cells were then washed with DPBS 3 times to remove the surplus dye. The supernatant was discarded after centrifugation, and the precipitate was dissolved in 200 μl of DPBS. Cell apoptosis was observed within an hour of its measurement with flow cytometry.

### Fabrication of the cellular model

A hyperosmotic (HS) damaged HCEC model was prepared for use in this work (Meng et al., 2021). HCEC cells were incubated with different NaCl solutions for 24–72 h, which created different osmotic pressures. The culture medium with different osmotic pressures ranging from 310 to 600 mOsm was applied. HCEC cells under a 500 mOsm pressure for 4 h were used as the HS cellular model in this work.

### *In vivo* experiments

20 adult male BALB/C mice (aged 3–6 months, at a weight of 20–25 g) were divided into four groups. These include the negative control group (Control), the dry eye model group, the PS treated HS dry eye group (PS), and the PS-CG treated dry eye group (PS-CG). The Control was treated with 0.9% normal saline while the other three groups were treated with 0.1% benzalkonium chloride (Clouzeau et al., 2012; Chen et al., 2021). Benzalamine chloride was topically administered into the eyes of experimental mice to create the dry eye animal model. All four groups were treated with eye drops twice a day over 14 days. PS or PS-CG was tested as a treatment for this model.

An epithelial injury refers to the formation of a dry eye model, which was observed using a slit-lamp. 0.9% normal saline was applied to a control and the dry eye group continuously once a day for an additional 3 weeks. 4 μg/ml PS solution or PS-CG dispersion was applied to the PS and the PS-CG group, respectively. Tears were collected with a capillary glass tube from the lower fornix of the experimental mouse in their natural state.

The mice thus tested were put to sleep at the end of the experiment. Their corneas, the clear part of the eye, were removed and put into a 4% paraformaldehyde liquid that preserves body tissue. Pruning, dehydration, embedding, sectioning, staining and sealing were part of this process for the pathological examination of the mice. The standard operation process (SOP) was followed strictly for these procedures. The researchers looked at the samples stained with HE under a microscope and took pictures. An Image-Pro Plus 6.0 program was used for image analysis.

### Ethical conduct of research

The animal experimental procedures performed for this work were approved by the Wenzhou Medical University Animal Care and Use Committee. These experiments followed the ethical principles for medical research outlined in the Declaration of Helsinki documents.

### Statistical analysis

A one-way analysis of variance (ANOVA) and a posthoc test were calculated to compare means between two or more groups. SPSS 16.0 software (Version 23, Armonk, United States) was used to calculate any statistical difference. Experiment data collected for statistical analysis was done at least in triplicate. A *p < 0.05 indicated a significant difference, A **p < 0.01 represented a high significance, and a ***p < 0.005 suggested a very high significance.

## Results and discussion

### Characterization

**FIGURE 1:**
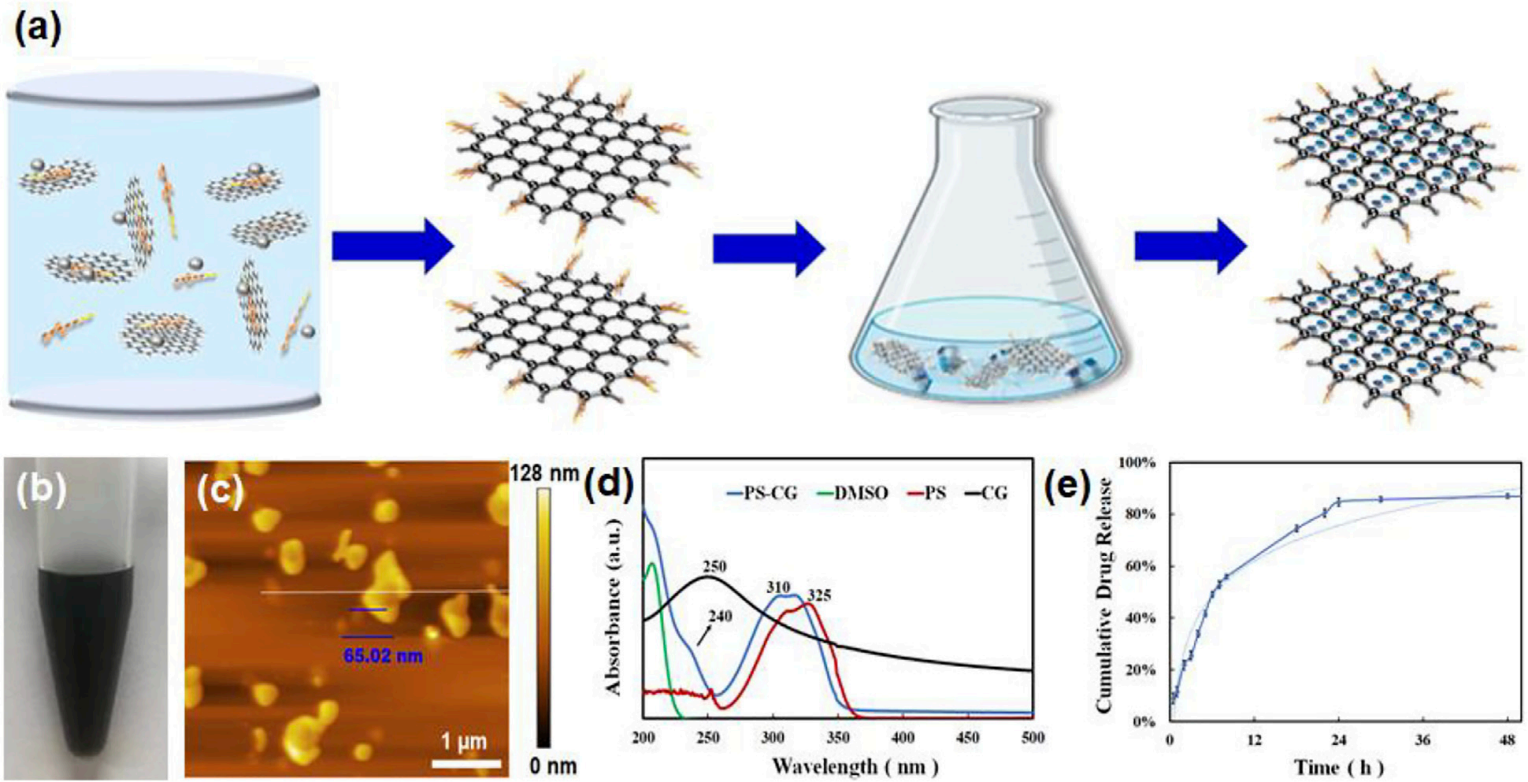
**(A)** A schematic illustration of the synthesis of PS-CG nanocomposite; **(B)** The dispersion of PS-CG in DI water; **(C)** AFM micrograph of the as-prepared PS-CG; **(D)** UV-vis spectrum of PS-CG, compared that of the pristine PS, CG and DMSO, respectively; **(E)** The release profile of PS from the PS-CG nanocomposite.

The carboxyl-chitosan modified graphene (CG) was fabricated *via* an edge-functionalized ball milling method. The process is shown in Figure 1A. The solid-state mechanochemical reaction was started by grinding a mixture of carboxyl-chitosan and graphite powder (Jeon et al., 2013). This caused the functionalization of the chitosan on the edge of graphite sheets, giving rise to the breaking of covalent bonds which connect the graphite layers and the formation of graphene nanosheets (Zou et al., 2020). The PS was covalently linked to CG *via* a π-π stacking interaction. Figure 1B shows that the as-prepared PS-CG is good dispersible in DI water. A uniform dispersion can remain intact in the air over 4 weeks.

This work found that adding CG to PS made it more dispersible in water. An atomic force microscopy (AFM) image in Figure 1C showed that PS-CG had a flat, nanoflake-like structure. The average thickness of the PS-CG nanosheet was about 65 nm. Supplementary Figure S1 shows the Raman spectrum of CG and compares it to that of the pristine graphite and carboxyl-chitosan. The Raman spectrum of CG shows three peaks: a D band at 1,349 cm^−1^, which is associated with the edge defects i.e., the sp^3^-hybridized carbon; a G band at 1,582 cm^−1^, which is related to the highly ordered graphite such as sp^2^ carbon, and a 2D band at 2,673 cm^−1^, which can be attributed to a two phonon interval double resonance scattering. This suggests the presence of graphene nanosheets. Compared to the spectrum of the pristine graphite, the peak intensity ratio of the D band to the G band (I_D_/I_G_) on CG increased, meaning that there were more defects (i.e., chitosan) in the structure of the graphite matrix after the mechanochemical reaction was performed.

After incorporation of PS with CG *via* a π-π stacking interaction, the PS-CG aqueous dispersion was characterized using various techniques. As shown in Figure 1D, the UV-vis spectrum of PS-CG has the characteristic peaks of both PS (at 310 nm) and CG (at 240 nm). The characteristic peak of the pristine PS was blue-shifted from 325 to 310 nm after PS was immobilized onto the CG carrier. This supports a π-π stacking interaction between PS and CG (Huo et al., 2007). When compared to pristine PS and CG, PS-CG has a different Fourier transform infrared (FTIR) spectrum as seen in Supplementary Figure S2. The typical characteristic peaks of CG were observed. A broad band at 3,438 cm^−1^ is due to the overlapped stretching of vibration of N-H and O-H. Two peaks at 1,639 and 1,394 cm^−1^ are due to the presence of carboxyl-functional groups. A peak at 1,078 cm^−1^ is associated with the stretching vibration of C-N. These peaks are visible in the spectrum of the as-synthesized PS-CG (as indicated by the red arrows in the blue curve). Four characteristic peaks due to the presence of the aromatic ring were found in the spectrum of PS (the red curve) at 1,620, 1,590, 1,530, and 1,430 cm^−1^, respectively. The peak at 3,423 cm^−1^ is attributed to the stretching vibration of O-H. These peaks occurred in the spectrum of PS-CG (as indicated by the black arrows in the blue curve). FTIR results confirmed that PS-CG was successfully synthesized.

Supplementary Figure S3 shows the changes in the colors that UV-vis spectra of PS absorb at various concentrations. The most absorption happens at 325 nm. A standard curve is a graph that shows how two things are related. In this case, it shows how the UV absorption intensity (how much light is absorbed) at 325 nm (a wavelength of light) is related to the concentration of PS that was obtained in Supplementary Figure S3 and shown in Supplementary Figure S4. A standard curve is used to calculate the concentration of PS-CG in a sample by looking at how much light is absorbed in 325 nm. The release profile of PS from PS-CG is shown in Figure 1E. A total of 55% PS was released after 8 h. This suggests the physical absorbed PS accumulates onto the surface of PS-CG, and it is easy to release. After 8 h, the release of PS is gradual. After 30 h, 85% of PS has been released.

### *In vitro* antioxidant performance

**FIGURE 2:**
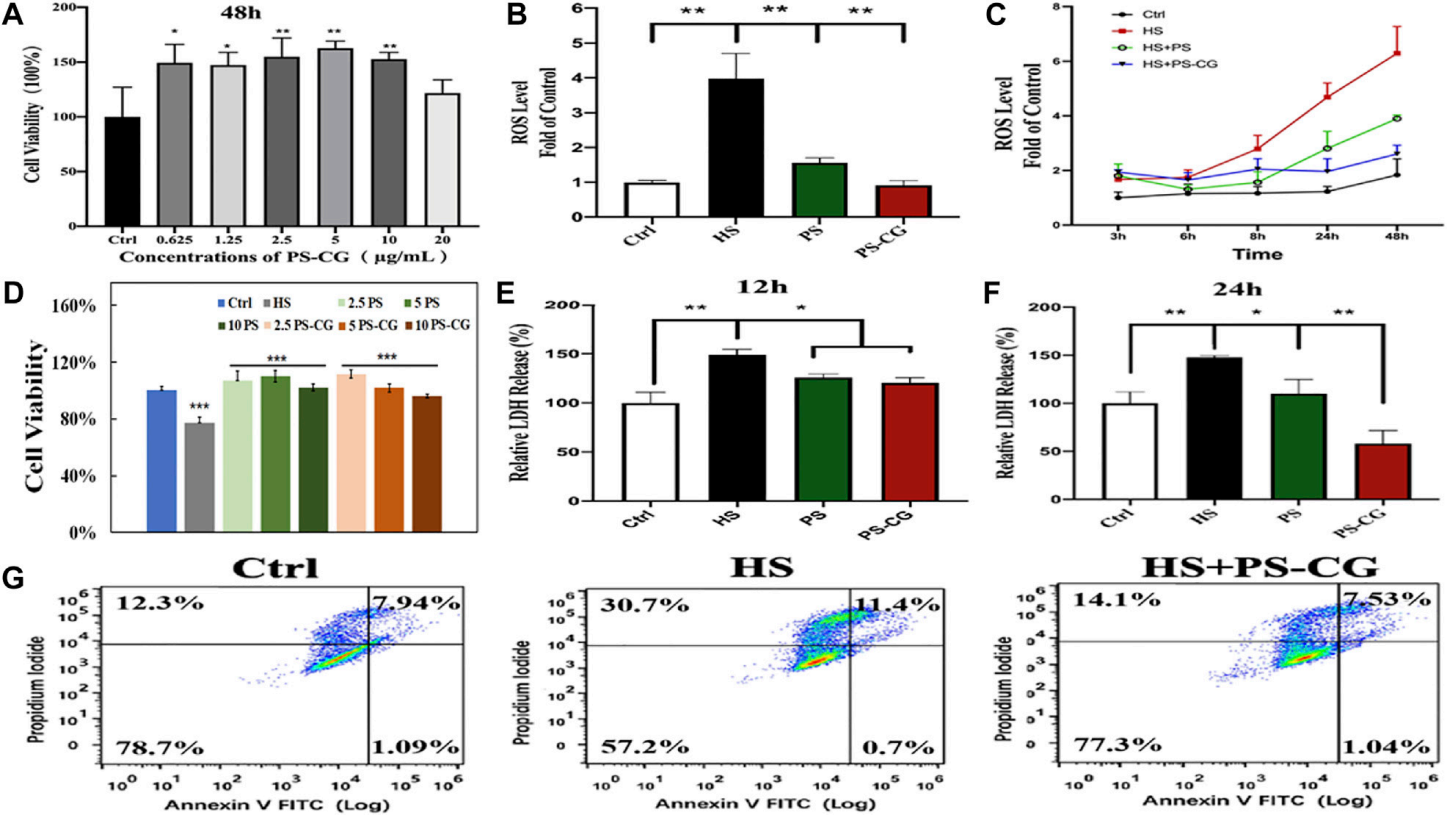
**(A)** Cell viability of HCEC cells co-incubated with PS-CG over 48 h; **(B)** The ROS level at the hyperosmotic (HS) model with the treatment of PS or PS-CG over 24 h, respectively; **(C)** The variability of the ROS level at the HS model after using PS or PS-CG for various periods; **(D)** the changes of the cell viability at the HS cellular model after treatment with different amounts of PS or PS-CG (at a concentration from 2.5 to 10 μg/ml) over 24 h; **(E,F)** The content of the LDH release after the treatment on the HS model with PS or PS-CG over 12–24 h; **(G)** Flow cytometry results regarding the cell apoptosis of the HS cellular model after the treatment of PS-CG. Bar errors as shown indicate standard errors. A *p < 0.05 indicates a significant difference. A **p < 0.01 represents a high significance.

The biocompatibility of the as-synthesized PS-CG against HCEC cells was tested using a CCK-8 cell viability assay. Up to 20 μg/ml, PS-CG was co-cultured with HCEC over 48 h. As shown in Supplementary Figure S5; Figure 2A, the cell survival rate increased by more than 50% after the introduction of fewer than 20 μg/ml PS-CG. This suggests that the PS-CG is good for the cells and helps them grow. Different concentrations of NaCl salt were used to create various osmotic pressures for cells in the HS cellular models.

Supplementary Figure S6 shows the influence of osmotic pressure on the amount of ROS generated within HCEC cells. The best enhancement effect on ROS was obtained when 500 mOsm was applied for 4 h. The cell survival rate of the HS cellular model was also optimal at around 80% when 500 mOsm was applied for 4 h as shown in Supplementary Figure S7. This suggested that these conditions were optimal and were used in this work for other *in vitro* tests. Supplementary Figure S8 shows the fluorescence micrograph of the DCFH-DA probe marked HS model after the treatment of PS-CG. The intensity of the green fluorescence indicates the content of ROS within cells. The fluorescent results were quantitatively illustrated in Figure 2B. As can be seen, the ROS content was increased by about 4 times in the HS model compared to the control group. When PS was added to the HS model over 24 h, the ROS level significantly decreased. This suggests the good antioxidant capacity of the pristine PS. But the ROS level was 50% higher than the control group. When PS-CG was applied, the ROS level went back to normal after 24 h. This supports the superior antioxidant capacity of PS-CG. When the treatment time was increased to two days, there was a significant increase in the ROS level for the PS treated HS model as shown in Figure 2C. The PS-CG treated HS model kept the ROS level at normal, suggesting that the PS-CG nanocomposite is good at protecting HCEC cells from oxidative stress and dry eye disease over the long term. The cell viability results are shown in Figure 2D suggest that both PS and PS-CG are useful for stimulating the growth of HCEC cells that were damaged in the HS model. Thus PS and PS-CG (less than 10 μg/ml) is not toxic to HCEC cells and can help them to grow.

The increased level of ROS in the HS model damaged cell membranes and caused the rising expression of lactate dehydrogenase (LDH) as shown in Figures 2E,F. In the HS model, a 150% LDH content was observed when compared to the control group. We found that PS and PS-CG decreased the levels of LDH within 12 h. The level of LDH in the both PS and PS-CG treated HS model decreased to 120% of the level seen in the Control after 12 h. The LDH content in the PS treated HS model remained similar when the treatment time was extended to 24 h. But the LDH level at the PS-CG treated model continuously declined to less than that of the control. This further confirms that PS-CG was good at preventing cell damage over a long period. An Annexin V-PI apoptosis detection kit was used to evaluate cell apoptosis using flow cytometry. As shown in Figure 2G, there were a total of four quadrants in the result of flow cytometry. The ratio of viable cells is shown in the lower-left corner of the four quadrants. After 36 h, there are 57.2% viable cells in the HS model. This is a 20% decrease in viable cell numbers when compared to the control group, which has 78.7% viable cells. When the HS model was treated with PS-CG over 36 h, the percentage of cells that were still alive increased to 77.3%. The upper left quadrant represents the percentage of necrotic cells and a small percentage of cells that underwent apoptosis at the late stage. In the upper right quadrant, the ratio of the primary apoptotic cells at the late stage is shown. In the HS group after 36 h, 30.7% necrotic cells and 11.4% apoptotic cells were observed. This is a significant increase when compared to those seen in the control group (i.e., 12.3% necrotic cells and 7.94% apoptotic cells, respectively). The oxidative stress in the HS model caused worse biological damage including cell necrosis and cell apoptosis. After PS-CG was added to the HS model, the ratio of necrotic cells declined to 14.1% while the proportion of apoptotic cells declined to 7.53%. PS-CG is an excellent candidate as an antioxidant.

### Antioxidant mechanism investigation

**FIGURE 3:**
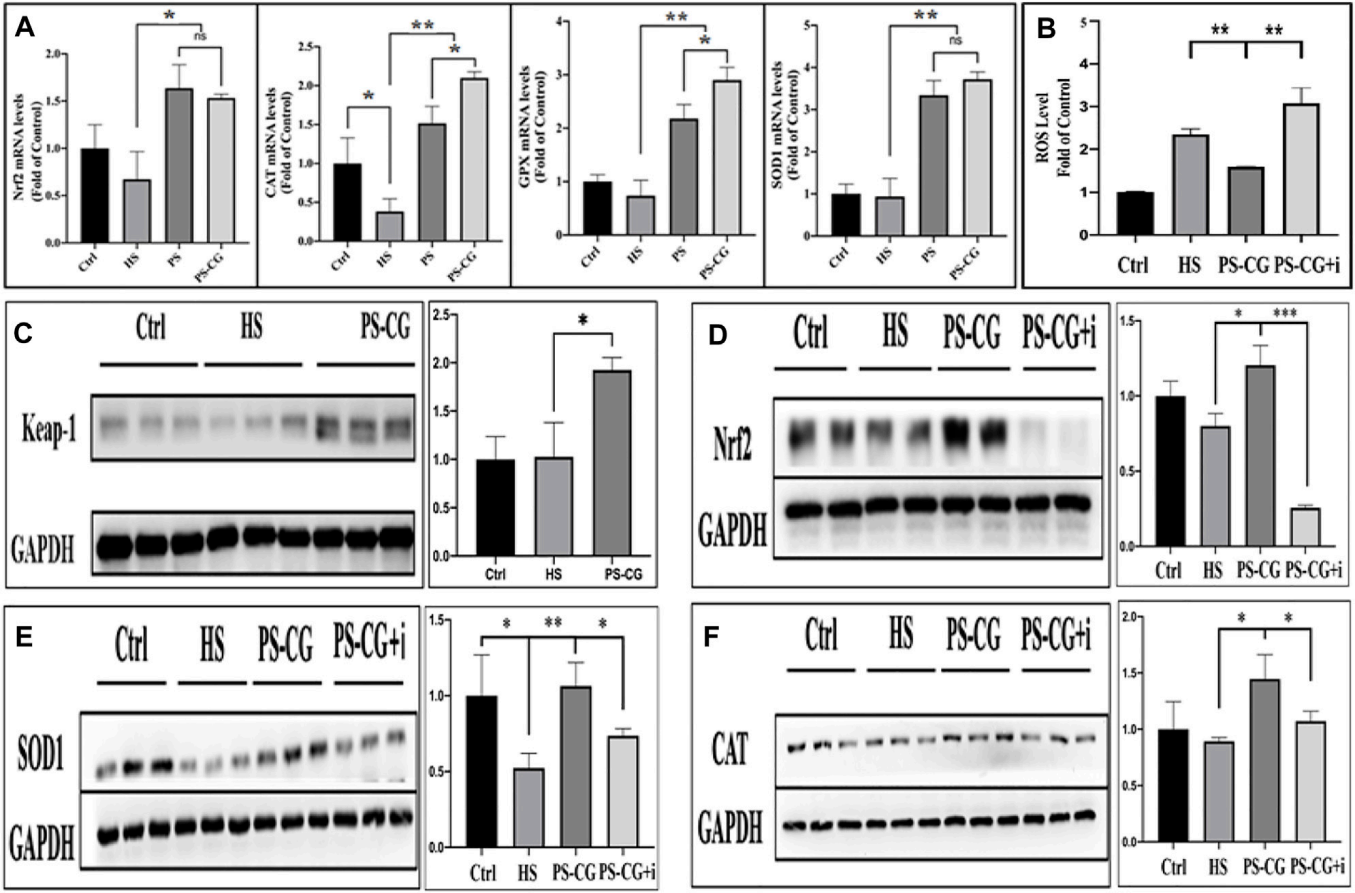
**(A)** The real-time quantitative PRC measurement of the expression of antioxidative stress-related proteins including Nrf2, CAT, GPX, and SODI, respectively, after the HS cellular model was treated with PS or PS-CG; **(B)** Changes in the ROS content after the treatment on the HS model with PS-CG or PS-CG plus Nrf2 inhibitor (PS-CG + I; **(C–F)** A typical Western blots bands and their quantitative analysis regarding the Keapl-Nrf2-ARE antioxidative stress signalling pathway-related proteins such as Keap-1, Nrf2, SOD1, and CAT, after the HS cellular model was treated with PS-CG, or PS-CG plus the corresponding protein inhibitors. Bar errors indicate the standard errors. A *p < 0.05 indicates a significant difference. A **p < 0.01 represented a high significance, and a ***p < 0.005 suggests a very high significance.

A real-time quantitative PCR was used to study the changes of proteins at the molecular level to better understand the antioxidant mechanism of PS-CG. Four anti-oxidative stress-related proteins (Nrf2, CAT, GPX and SOD1) were selected for mRNA expression and analysis. In Figure 3A, it can be seen that the expressions of anti-oxidative stress-related proteins (such as Nrf2, CAT, GPX and SOD1) are significantly enhanced after treatment of the HS model with PS or PS-CG over 24 h. This suggests that the antioxidant effect of PS and PS-CG may be realized by the activation of antioxidative stress-related pathways. This work found that the CAT and GPX proteins are expressed more in the PS-CG group than in the PS group. No difference was seen in the expressions of Nrf2 and SOD1 between the two groups. The Keap1-Nrf2-ARE signalling pathway is an important way for cells to protect themselves from oxidative stress injuries (Battino et al., 2018). These results showed that the expression of Nrf2 increased a lot after the cells were treated with PS-CG. The antioxidant mechanism of PS-CG may be related to this pathway. We then tested the expression of proteins associated with this pathway *via* a western blot (WB) assay.

No difference was seen in the expression of Keap1 and Nrf2 proteins between the HS group and the control group as shown in Figures 3C,D. This suggests that the oxidative stress damage in the HS model is caused by a strengthened oxidative stimulation and not by an inhibited antioxidant pathway. This means that after the treatment of PS-CG onto an HS model, the proteins Keap1 and Nrf2 were observed to be highly up-regulated. This change was also observed in the result of SOD1 and CAT (Figure 3E,F). Brusatol, an inhibitor of Nrf2, was then applied to the PS-CG treated HS model; Nrf2 expression decreased sharply as shown in Figure 3D. When the Nrf2 inhibitor was applied, there was a significant decline in the expression of two antioxidant enzymes, SOD1 and CAT (Figure 3E,F). This confirmed that the proteins of SOD1 and CAT are the downstream proteins of Nrf2 in the Keap1-Nrf2-ARE signalling pathway. Thus the efficient antioxidant effect of PS-CG might be caused by the up-regulation of Nrf2, which leads to the enhanced expression of antioxidant enzymes.

To test this hypothesis, we looked at the content of ROS after using the Nrf2 inhibitor. We found that an increase in ROS in the HS model was efficiently inhibited by using PS-CG as shown in Figure 3B. The Nrf2 inhibitor was applied to the PS-CG treated HS model, and the ROS level re-ascended sharply. This suggests that the inhibition of Nrf2 blocks the Keap1-Nrf2-ARE antioxidant signalling pathway, and as a result, PS-CG is not effective in improving the antioxidant performance. It is thus likely that the antioxidant effect of PC-CG is achieved through activation of the Keap1-Nrf2-ARE pathway.

### *In vivo* study on a dry eye mouse model

**FIGURE 4:**
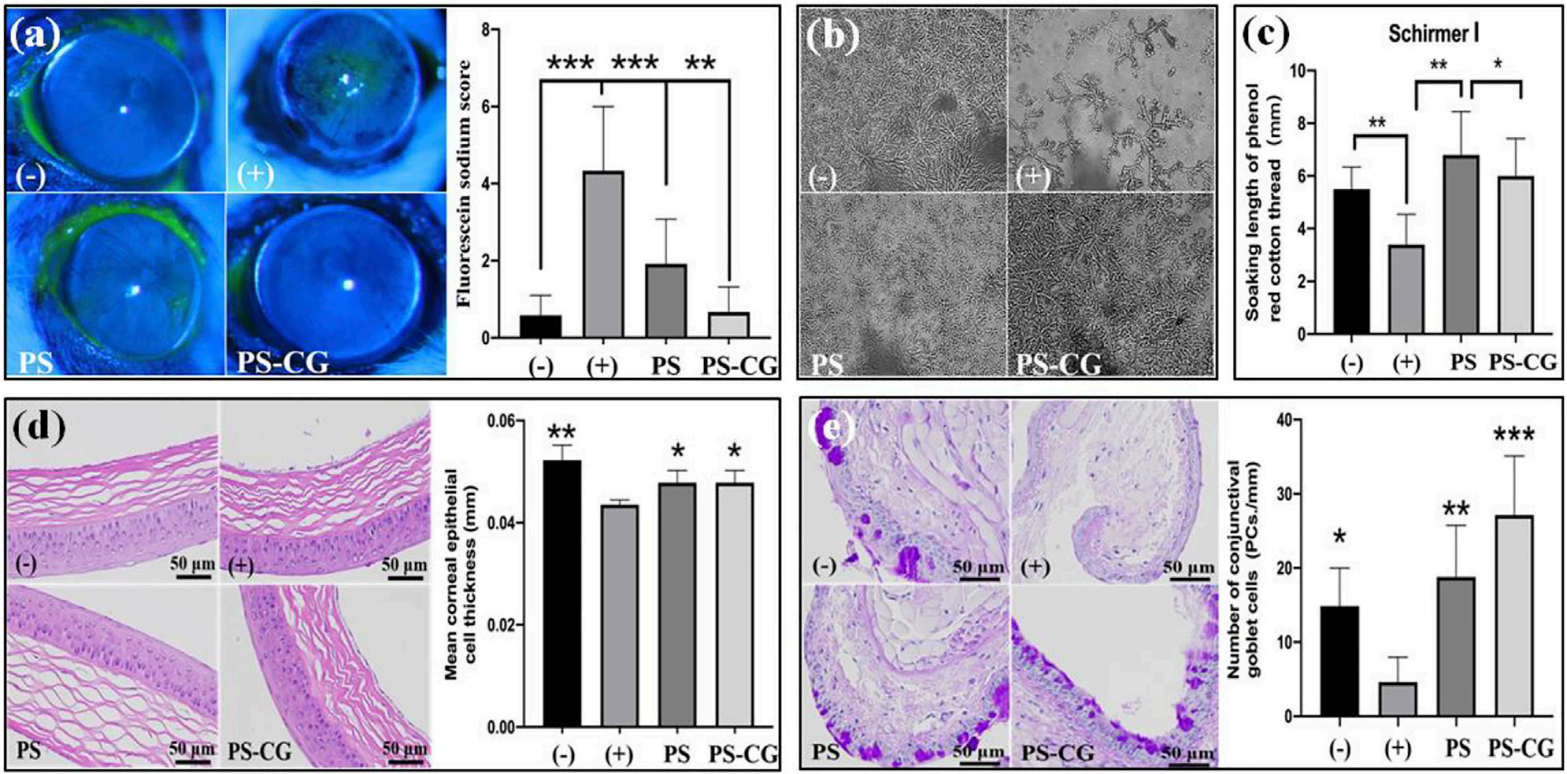
**(A)** Fluorescein sodium stained cornea surfaces of the experimental mice and the quantitative analysis; **(B)** Crystallization experiment of tear ferns from the experimental mice; **(C)** Tear secretion measurement; **(D)** HE stained corneal epithelium and its thickness measurement; **(E)** conjunctival PAS staining and goblet cell count results. Bar errors indicate the standard errors. A *p < 0.05 indicates a significant difference, A **p < 0.01 represented a high significance, and a ***p < 0.005 suggests a very high significance.

Corneal fluorescein staining can show problems with the surface of the eye. A healthy eye should not have any fluorescence, but if there is an ulcer or other defect, it will show up as a bright spot as shown in Figure 4A. The yellow-green fluorescence means that there is an inflammatory injury on the cornea. This kind of fluorescence was seen in the positive dry eye group (+), where around 30% distribution range was fluorescent. Almost five times as many corneal staining scores were achieved. The corneal fluorescence decreased significantly in the PS treated dry eye group, even though a small amount of fluorescence was still visible. In this experiment, the staining score decreased by 50% when PS was applied to the dry eye group. When PS-CG was applied, the staining score decreased even more, to the level seen in the negative control group.

Corneal epithelial injury is a common problem for people with dry eyes. There is not enough tear secretion and the composition of tears is different. A crystallization experiment of tear ferns and a tear secretion measurement using phenolic cotton threads were thus carried out. The crystallization measurement on tear ferns reflected the compositional change of tear, especially the content of mucin. Dry eyes happen when there’s not enough mucin in tears. Mucin is a sticky substance that helps keep the ocular surface moist. Normally, mucin is in a gel state. But when it is reduced significantly, the surface of the eye can get dry and irritated (Baudouin et al., 2019). The gel helps protect the surface of the eye. When there is not enough of it, the epithelial surface can’t be repaired as well.

The negative control group had normal tears with well-formed ferns that were uniformly distributed and had numerous branches as shown in Figure 4B. The dry eye group’s ferns crystalline materials almost disappeared. The number of ferns in the PS treated dry eye group increased significantly. The ferns were distributed evenly throughout the field of vision. But the number and length of branches did not recover to normal. The number and length of branches increased further when PS-CG was applied, which helps the tear composite recover back to its normal range. In the dry eye group, the amount of tear secretion declined by one third when compared to the control group as shown in Supplementary Figure S9; Figure 4C. However, after using PS or PS-CG, the tear secretion in the dry eye group increased back to its normal level.

The image in Figure 4D shows a close-up of the surface of the eye. In a healthy eye, the epithelium is smooth and evenly stratified. The corneal epithelium is made up of 4–5 layers of cells. The basal cells are the cells that form the bottom layer of the epithelium. The basal cells are shaped like columns. They gradually change into squamous epithelial cells as they move up towards the surface of the cornea. This work found that the injected benzalamine chloride caused some basal cells to disappear and the thickness of the corneal epithelium to decline. The thickness of corneal epithelium decreased significantly from 52 µm in the control group to 43 µm for the dry eye group (where *p* < 0.01). The thickness of the cornea recovered to 48 µm after the treatment with PS or PS-CG. Mucins are substances that are secreted from the conjunctival goblet cells and corneal epithelial cells. The number and state of goblet cells in the conjunctiva affect the secretion of mucin.

Figure 4E shows the difference in PAS stained conjunctiva and the number of goblet cells between the control group and the test group. The control group had a good tissue structure with multiple stained goblet cells distributed. Atrophy of the conjunctival tissue without visible goblet cell distribution, however, was observed in the HS group. After its treatment of PS or PS-CG in the dry eye group, the number of goblet cells increased sharply and there was a good conjunctival tissue structure. A reduction of nearly 70% was seen in the number of goblet cells per unit length in the dry eye group when compared to the negative control group. However, when PS was applied to the dry eye group, the number of goblet cells increased rapidly to 127% of the normal level obtained in the negative control group. The number of goblet cells per unit length in the PS-CG group further increased by 183% from the normal level. In a typical dry eye model, the stability and lubrication of the corneal surface area are affected by conjunctival tissue atrophy, goblet cell injury and mucin secretion decline, and this results in damage to the corneal epithelium. Particularly, the significantly reduced amount of tear secretion causes further damage to the corneal epithelium. The new PS-CG helps to protect cells in the cornea and conjunctiva from oxidative stress damage, beneficial in the recovery of the tissue function and the tear secretion improvement. This helps to repair the damaged corneal epithelium, providing efficient treatment for dry eye diseases.

Supplementary Figure S10 shows the results of a test to see how safe PS-CG is for mice. It shows the HE stained pathological section of important organs such as the heart, liver, spleen, lung, and kidney, taken from the PS-CG treated mouse model. It is found that there were no harmful effects. Thus the PS-CG does not cause any visible changes in organs when compared to organs that were not exposed to the PS-CG.

## Conclusion

This work reports the development of a new type of antioxidant, made from a PS-CG nanocomposite, for the treatment of dry eye disease. A two-dimensional carboxyl-graphene was used as the nanocarrier for the immobilization of antioxidant PS. The thus fabricated PS-CG had a typical nanoflake shape with an average thickness of 65 nm. Good biocompatibility and water dispersibility of the as-synthesized PS-CG were obtained. The PS was covalently linked with CG *via* a π-π stacking interaction. This enabled a stable sustained release of PS from PS-CG. An HS injury HCEC cell model was used to test how well an antioxidant could reduce oxidative stress damage. The antioxidant worked well, efficiently reducing the increased ROS in the HS group to normal levels. The cell membrane was damaged and the cell viability declined in the HS model, but this became normal after treatment with PS-CG. The number of apoptotic and necrotic cells increased in the HS model but significantly declined back to normal by using PS-CG. These results suggest that PS-CG is useful in the protection of cells from oxidative stress damage. Quantitative real-time PCR and WB results show that it activates the Keap1-Nrf2-ARE signalling pathway, which is an antioxidant pathway. The expression of various downstream antioxidant enzymes suggests that the cells are creating more antioxidants. In a dry eye animal model, the application of PS-CG was found to make the tears secrete more, and also to make the corneal and conjunctival epithelial cells healthier. Using PS-CG on a dry eye model increased the number of goblet cells and the production of mucin and increased tear secretion. This work observed that the PS-CG nanocomposite was effective in treating dry eye disease and could provide a novel solution for the treatment of ocular diseases related to ROS.

## Data Availability

The original contributions presented in the study are included in the article/Supplementary Material, further inquiries can be directed to the corresponding authors.
