# Supplementary material for: A new antioxidant made from a pterostilbene functionalized graphene nanocomposite as an efficient treatment for dry eye disease
Source: Front Chem. 2022 Aug 24;10:942578. doi: 10.3389/fchem.2022.942578 (PMC9449147; doi:10.3389/fchem.2022.942578)
Supplement: Supplementary file 1 [file DataSheet1.docx]

**Supplementary Material**

**A new antioxidant made from a pterostilbene functionalized graphene nanocomposite as an efficient treatment for dry eye disease**

**Mimi Lin^1,2,#^, Xueqin Sun^1,2,#^, Sihao Ye^1,2^, Youyi Chen^1,2^, Jing Gao^1,2^, Feng Yuan^1,2^, Na Lin^2^, Tom Lawson^3^, Yong Liu^1,2,^*, and Ruzhi Deng^1,2,^***

^1^Laboratory of Nanoscale Biosensing and Bioimaging (NBAB), School of Ophthalmology and Optometry, School of Biomedical Engineering, Wenzhou Medical University, 270 Xueyuanxi Road, Wenzhou City, Zhejiang 325027, China

^2^Eye Hospital, State Key Laboratory of Ophthalmology, Optometry, and Vision Science, Wenzhou Medical University, Wenzhou City, Zhejiang 325027, China

^3^School Mathematical and Physical Sciences, ARC Centre of Excellence for Nanoscale Biophotonics (CNBP), Macquarie University, Sydney, NSW 2109, Australia
^#^These authors contributed equally to this work.

*Correspondence: yongliu@wmu.edu.cn (Y.L.); drz@eye.ac.cn (R.D.)


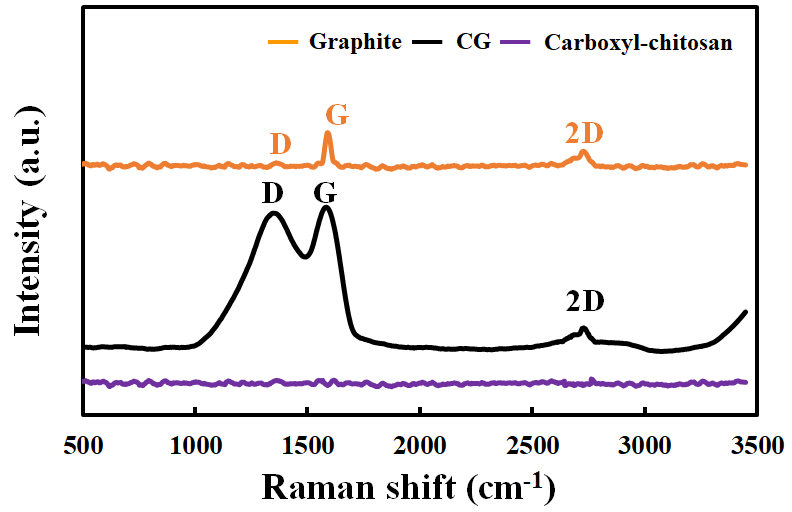


**FIGURE S1** Raman spectrum of the as-prepared CG, compared to the pristine graphite and carboxyl-chitosan.


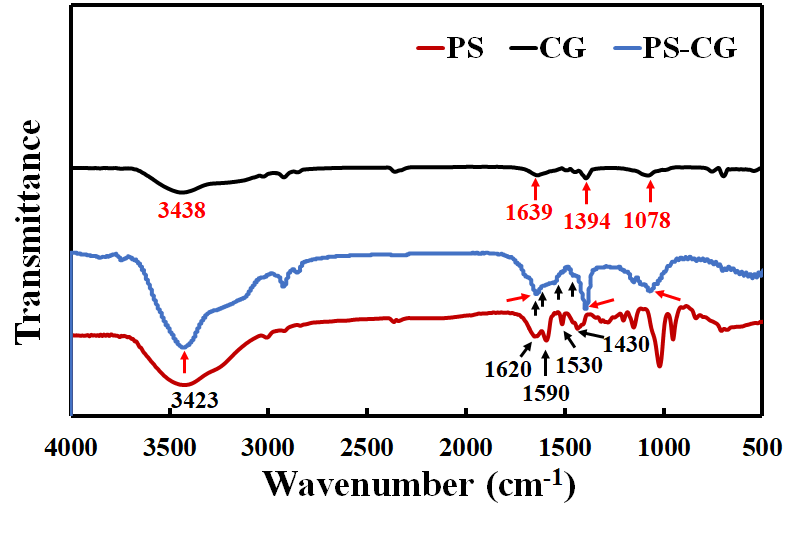


**FIGURE S2** FTIR spectrum of the as-prepared PS-CG, compared to the pristine PS and CG.


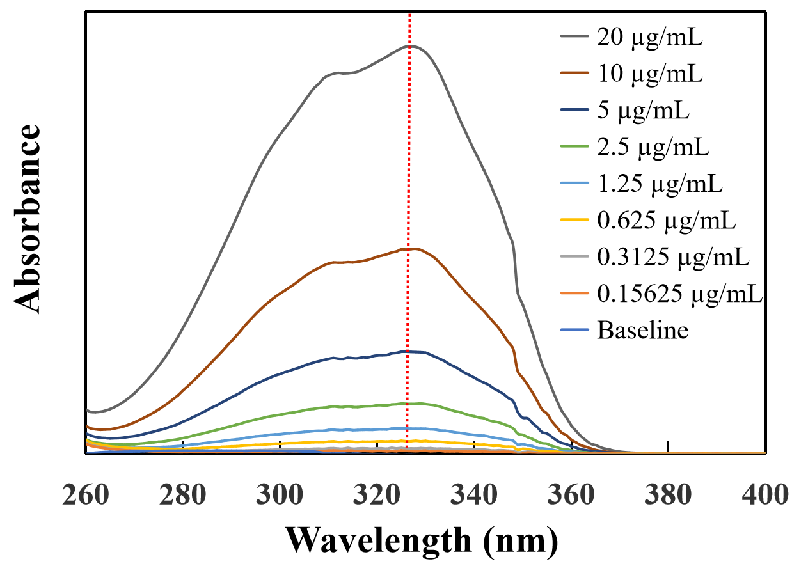


**FIGURE S3** (a) UV absorption spectra of PS at different concentrations. A significant absorption peak of PS is seen at 325 nm wavelength.


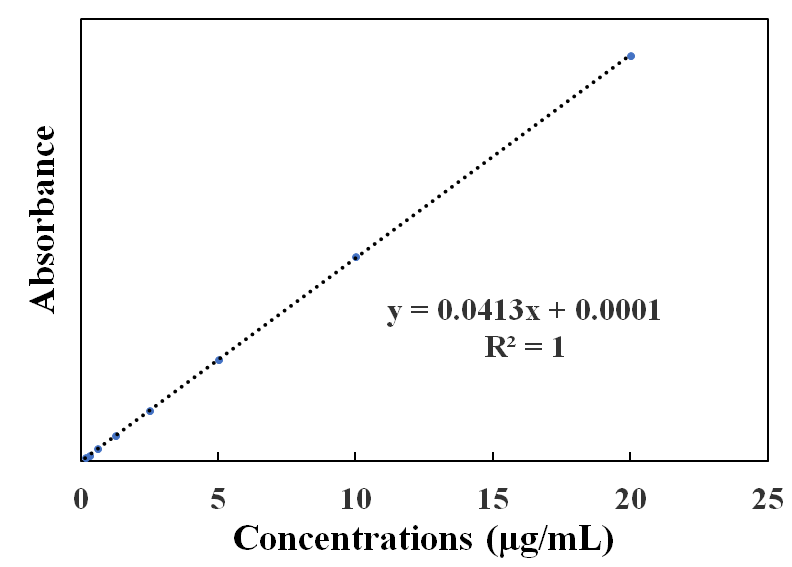


**FIGURE S4** The standard curve of the UV absorption intensity at 325 nm *VS.* the PS concentration.


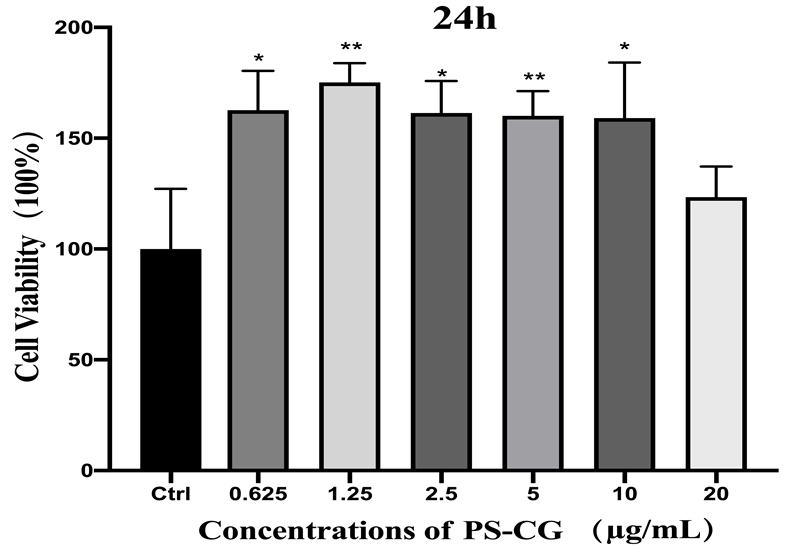


**FIGURE S5** Viability of HCEC cells after co-incubation with PS-CG over 24 hrs. Normal cells without hypertonic stimulation were used as the negative control. Bar errors indicate the standard errors. A *p < 0.05 indicates a significant difference. A **p < 0.01 represents a high significance.


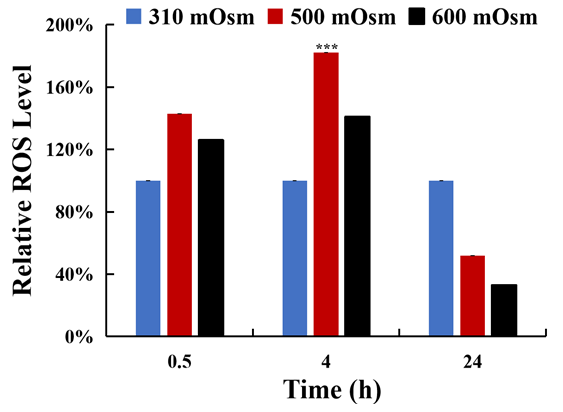


**FIGURE S6** The content of ROS inside the HCEC cellular model after employment of different osmotic pressures over various culture time. Bar errors indicate the standard errors. A ***p < 0.005 suggests a very high significance.


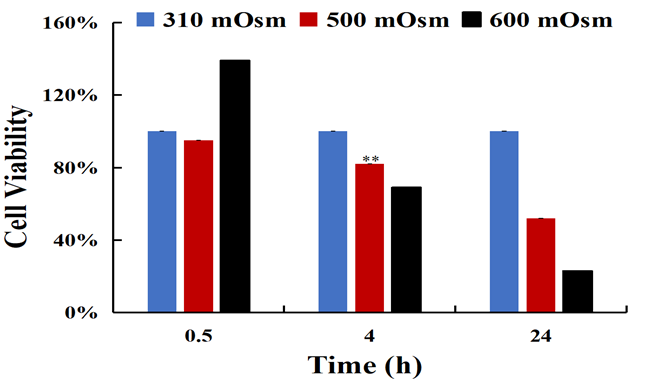


**FIGURE S7** Cell viability of the HCEC cellular model after employment of different osmotic pressures over various culture time. Bar errors indicate the standard errors. A **p < 0.01 represents a high significance.


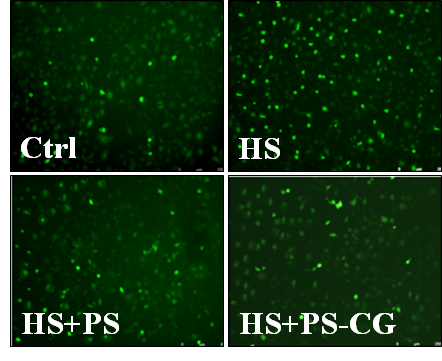


**FIGURE S8** Fluorescent micrographs of the DCFH-DA probe marked HS model after the treatment of PS or PS-CG.


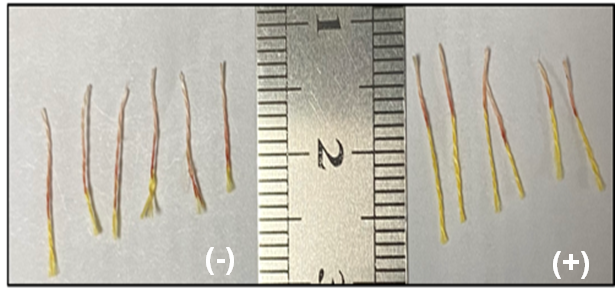


**FIGURE S9** Digital photos of the phenolic cotton threads after the tear secretion measurement. The left side shows the threads after measurement from the negative Control. The right side indicates the threads after measurement from the positive dry eye group. (n=6)


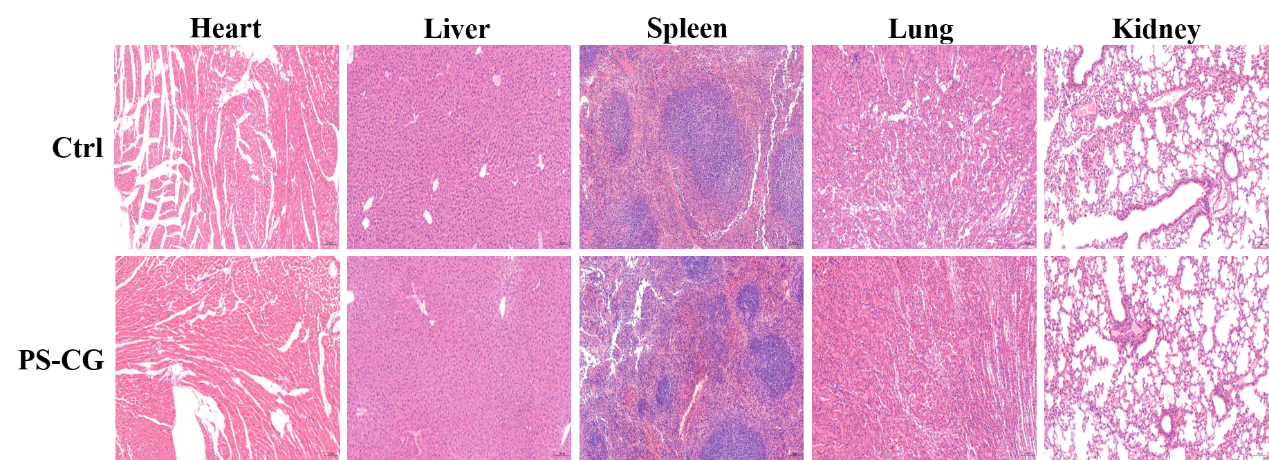


**FIGURE S10** Micrographs of HE stained pathological sections of heart, liver, spleen, lung, and kidney taken from the PS-CG treated dry eye mice.
